# Guidelines for Perioperative Care for Emergency Laparotomy Enhanced Recovery After Surgery (ERAS) Society Recommendations: Part 1—Preoperative: Diagnosis, Rapid Assessment and Optimization

**DOI:** 10.1007/s00268-021-05994-9

**Published:** 2021-03-06

**Authors:** Carol J. Peden, Geeta Aggarwal, Robert J. Aitken, Iain D. Anderson, Nicolai Bang Foss, Zara Cooper, Jugdeep K. Dhesi, W. Brenton French, Michael C. Grant, Folke Hammarqvist, Sarah P. Hare, Joaquim M. Havens, Daniel N. Holena, Martin Hübner, Jeniffer S. Kim, Nicholas P. Lees, Olle Ljungqvist, Dileep N. Lobo, Shahin Mohseni, Carlos A. Ordoñez, Nial Quiney, Richard D. Urman, Elizabeth Wick, Christopher L. Wu, Tonia Young-Fadok, Michael Scott

**Affiliations:** 1 Department of Anesthesiology and Gehr Family Center for Health Systems Science & Innovation, Keck School of Medicine, University of Southern California, 2020 Zonal Avenue IRD 322, Los Angeles, CA 90033 USA; 2 Department of Anesthesiology, University of Pennsylvania, 3400 Spruce St, Philadelphia, PA 19104 USA; 3 Department of Anesthesia and Intensive Care Medicine, Royal Surrey County Hospital, Guildford, Surrey, UK; 4 Sir Charles Gardiner Hospital, Hospital Avenue, Nedlands, WA 6009 Australia; 5 Salford Royal NHS Foundation Trust, Stott La, Salford, M6 8HD UK; 6 University of Manchester, Manchester, UK; 7 Hvidovre University Hospital, Copenhagen, Denmark; 8 Harvard Medical School, Kessler Director, Center for Surgery and Public Health, Brigham and Women’s Hospital and Division of Trauma, Burns, Surgical Critical Care, and Emergency Surgery, Brigham and Women’s Hospital, 1620, Tremont Street, Boston, MA 02120 USA; 9 Faculty of Life Sciences and Medicine, School of Population Health & Environmental Sciences, Guy’s and St Thomas’ NHS Foundation Trust, King’s College London, Division of Surgery & Interventional Science, University College London, London, UK; 10 Department of Surgery, Virginia Commonwealth University Health System, 1200 E. Broad Street, Richmond, VA 23298 USA; 11 Department of Anesthesiology and Critical Care Medicine, Department of Surgery, The Johns Hopkins University School of Medicine, 1800 Orleans Street, Baltimore, MD 21287 USA; 12 Department of Emergency and Trauma Surgery, Karolinska University Hospital, CLINTEC, Karolinska Institutet, Stockholm, Sweden; 13 Karolinska University Hospital, Huddinge Hälsovägen 3. B85, S 141 86, Stockholm, Sweden; 14 Department of Anaesthesia, Perioperative Medicine and Critical Care, Medway Maritime Hospital, Windmill Road, Gillingham, Kent, ME7 5NY UK; 15 Division of Trauma, Burns and Surgical Critical Care, Brigham and Women’s Hospital, 75 Francis Street, Boston, MA 02115 USA; 16 Department of Surgery and Critical Care Medicine, University of Pennsylvania, Philadelphia, PA 19104 USA; 17 Department of Visceral Surgery, Lausanne University Hospital CHUV, University of Lausanne (UNIL), Rue du Bugnon 46, 1011 Lausanne, Switzerland; 18 Gehr Family Center for Health Systems Science & Innovation, Keck School of Medicine, University of Southern California, 2020 Zonal Avenue IRD 322, Los Angeles, CA 90033 USA; 19 Department of General & Colorectal Surgery, Salford Royal NHS Foundation Trust, Scott La, Salford, M6 8HD UK; 20 Department of Surgery, Faculty of Medicine and Health, School of Health and Medical Sciences, Örebro University, Örebro, Sweden; 21 Gastrointestinal Surgery, Nottingham Digestive Diseases Centre and National Institute for Health Research (NIHR) Nottingham Biomedical Research Centre, Nottingham University Hospitals and University of Nottingham, Queen’s Medical Centre, Nottingham, NG7 2UH UK; 22 MRC Versus Arthritis Centre for Musculoskeletal Ageing Research, School of Life Sciences, University of Nottingham, Queen’s Medical Centre, Nottingham, NG7 2UH UK; 23 Division of Trauma and Emergency Surgery, Department of Surgery, Orebro University Hospital & School of Medical Sciences, Örebro University, 701 85 Örebro, Sweden; 24 Division of Trauma and Acute Care Surgery, Department of Surgery, Fundación Valle del Lili, Cra 98 No. 18 – 49, 760032 Cali, Colombia; 25 Sección de Cirugía de Trauma Y Emergencias, Universidad del Valle – Hospital Universitario del Valle, Cl 5 No. 36-08, 760032 Cali, Colombia; 26 Department of Anesthesia and Intensive Care Medicine, Royal Surrey County Hospital, Egerton Road, Guildford, Surrey, GU5 7XX UK; 27 Department of Anesthesiology, Perioperative and Pain Medicine, Brigham and Women’s Hospital / Harvard Medical School, 75 Francis Street, Boston, MA 02115 USA; 28 Department of Surgery, University of California San Francisco, 513 Parnassus Ave HSW1601, San Francisco, CA 94143 USA; 29 Department of Anesthesiology, Critical Care and Pain Medicine-Hospital for Special Surgery, 535 East 70th Street, New York, NY 10021 USA; 30 Department of Anesthesiology, Weill Cornell Medicine, 535 East 70th Street, New York, NY 10021 USA; 31 Division of Colon and Rectal Surgery, Department of Surgery, Mayo Clinic College of Medicine, Mayo Clinic Arizona, 5777 E. Mayo Blvd, Phoenix, AZ 85054 USA; 32 Department of Anesthesiology and Critical Care Medicine, University of Pennsylvania, 3400 Spruce St, Philadelphia, PA 19104 USA

## Abstract

**Background:**

Enhanced Recovery After Surgery (ERAS) protocols reduce length of stay, complications and costs for a large number of elective surgical procedures. A similar, structured approach appears to improve outcomes, including mortality, for patients undergoing high-risk emergency general surgery, and specifically emergency laparotomy. These are the first consensus guidelines for optimal care of these patients using an ERAS approach.

**Methods:**

Experts in aspects of management of the high-risk and emergency general surgical patient were invited to contribute by the International ERAS® Society. Pubmed, Cochrane, Embase, and MEDLINE database searches on English language publications were performed for ERAS elements and relevant specific topics. Studies on each item were selected with particular attention to randomized controlled trials, systematic reviews, meta-analyses and large cohort studies, and reviewed and graded using the Grading of Recommendations, Assessment, Development and Evaluation (GRADE) system. Recommendations were made on the best level of evidence, or extrapolation from studies on non-emergency patients when appropriate. The Delphi method was used to validate final recommendations. The guideline has been divided into two parts: Part 1—Preoperative Care and Part 2—Intraoperative and Postoperative management. This paper provides guidelines for Part 1.

**Results:**

Twelve components of preoperative care were considered. Consensus was reached after three rounds.

**Conclusions:**

These guidelines are based on the best available evidence for an ERAS approach to patients undergoing emergency laparotomy. Initial management is particularly important for patients with sepsis and physiological derangement. These guidelines should be used to improve outcomes for these high-risk patients.

## Introduction

Enhanced Recovery After Surgery (ERAS) is a multidisciplinary structured approach which provides standardized evidence-based components of care to patients undergoing specific types of surgery. To date, ERAS has largely been applied to elective surgery but there is now evidence that high-risk surgical patients such as those undergoing emergency laparotomy, can also benefit significantly from an ERAS approach [1–11]. The term “emergency laparotomy” encompasses a surgical exploration of the acute abdomen for a number of underlying pathologies [12–17]. Common causes are intestinal obstruction, perforation and exploratory laparotomy with or without wound debridement or abscess drainage [13–15, 17]. For these ERAS® Society guidelines the term “emergency” is applied to all patients with a non-elective, potentially life-threatening intra-abdominal condition requiring surgery, excluding trauma laparotomies, vascular conditions, appendectomy, and cholecystectomy.

Until recently patients undergoing emergency general surgery including emergency laparotomy have been a relatively overlooked group [15]. Just under a decade ago, major cohort studies reported 30-day mortality for emergency laparotomy of between 14 and 18.5% rising to over 25% in patients over 80 years of age [14, 18, 19]. A review of patients with advanced cancer who underwent emergency laparotomy for bowel perforation [20], showed a 30-day mortality of 34%, 52% of survivors were discharged to an institution. A number of studies have shown wide variation not only in outcomes, but also in the delivery of evidence-based care to this high-risk patient group [19, 21–28]. Given the concerning nature of these outcomes, namely high patient morbidity and mortality, a number of groups worldwide started using evidence-based protocolized ERAS-like approaches in the management of these patients, with significant improvements in outcomes [1–4, 6, 8, 29]. The UK established a National Emergency Laparotomy Audit (NELA), to measure process delivery and outcomes. Since the start of NELA data collection in 2013, outcomes have improved with 30-day mortality decreasing from 11.8% to 9.3% and performance on key process measures improving [17].

The important difference between patients undergoing emergency laparotomy and those undergoing elective intra-abdominal procedures is presentation of the former in a state of physiological derangement [13, 30]. Patients are often older [14, 17], have co-morbidities, and 30–50% present with systemic inflammatory response syndrome (SIRS), sepsis or septic shock [13, 14, 17, 30–33]. More emergency patients undergo an open procedure rather than a laparoscopic procedure for comparable surgery in the elective setting [5]. In spite of recent improvements, emergency laparotomy remains one of the highest risk surgical procedures with about one in ten patients deceased 30 days after surgery, rising to one in four over the age of 80 years [17]. Complications are common and mortality increases until at least 1 year [34]. Functional outcomes and return to independence can also be poor in survivors [35].

These high-risk patients are likely to benefit from a structured approach with defined pathways of care and organizational resource allocation to prioritize their management [26, 36]. As emergency laparotomy comprises a diverse group of patients and there are a number of new pathway components to be considered, we have divided these guidelines into Part One (preoperative care) and Part Two (intra- and postoperative care, organizational aspects of management, and end of life issues). We suggest these ERAS® Society Guidelines should be routinely applied to the care of patients undergoing emergency laparotomy and used to audit processes and outcomes of care.

## Materials and methods

This project was initiated by the ERAS® Society. Lead authors (CP and MS) were invited by the ERAS® Society to establish a guideline development group (GDG) of healthcare professionals with diverse expertise in the management of patients undergoing emergency laparotomy. The GDG consisted of surgeons, anesthesiologists, and geriatricians. A number of authors are accredited in intensive care, including the first and last authors, or have significant experience of intensive care management of these patients. The group was also selected to ensure international representation. A list of topics was generated and groups of physicians with different backgrounds and from different countries were assigned to each topic, based on their expertise, to perform a literature review of English language publications and then to generate recommendations using the GRADE structure [37]. The time period searched was from 2005 until mid-2020, with greater emphasis on recent publications, randomized controlled trials, systematic reviews, meta-analyses and large cohort studies. Retrospective studies were considered where no other higher level of evidence was available, and if there was particular relevance to emergency laparotomy. The guideline development and Delphi process [38] used to reach consensus on recommendations were based on the process published by the ERAS® Society [39]. Twelve components of preoperative care were considered. Three rounds of the Delphi process were performed.

### Definitions

In these guidelines, emergency laparotomy is defined in line with criteria used by large cohort studies [16, 40] and definitions of high-risk emergency general surgical procedures [41], therefore, trauma laparotomies, appendectomy, and cholecystectomy are excluded. The majority of vascular conditions are excluded such as laparotomy for vascular pathology including ruptured aortic aneurysm and return to theatre with complications following a vascular procedure. Conditions relating to bowel ischemia such as mesenteric vascular insufficiency are included [16, 40]. The definition of emergency can also vary, from classification of the case by the surgeon and anesthesiologist as an emergency [14, 42] to a definition used in a major US epidemiology study of emergency surgery [32] as non-elective surgery within 48 h of admission. NELA defines emergencies as patients having a non-elective admission with a potentially life-threatening condition [40], and urgency is defined [43] as immediate, urgent (surgery within hours of the decision to operate) or expedited (surgery within days of the decision to operate where some conservative management may occur initially). In these guidelines, the term “emergency” is applied to all patients with a non-elective, potentially life-threatening intra-abdominal condition requiring surgery.

### Commentary

The components of a standard elective colorectal pathway were reviewed in relation to the patient undergoing emergency laparotomy [44]. However, it is the additional management of the acute physiological derangement before, during and after surgery that leads to a unique emergency laparotomy pathway.

## Results

### Evidence and recommendations

A summary of the ERAS elements and grading of recommendations with their respective level of evidence are depicted in Table 1.

**Table 1:** ERAS Emergency laparotomy preoperative phase guideline review by delphi method [38, 39]

ERAS item	Guideline	Level of evidence	Recommendation grade
1. Early identification of physiological derangement and intervention	Resuscitation and correction of underlying physiological derangement should begin immediately and should continue during diagnostic pathways	High	Strong
	Rapid assessment of the patient for physiological derangement using a validated method such as an early warning scoring system should occur. Abnormal scores should trigger rapid escalation to senior personnel in line with pre-established local protocols. While awaiting surgery patients should have regular re-evaluation with a frequency dictated by local physiological track and trigger protocols	High	Strong
2. Screen and monitor for sepsis and accompanying physiological derangement	All patients for emergency laparotomy should be assessed with a validated sepsis score as early in their presentation as possible. This should be repeated at appropriate intervals in line with severity of signs, and sepsis risk stratification guidance	High	Strong
	If SIRs, sepsis or septic shock is diagnosed, treatment should begin immediately in line with the Surviving Sepsis recognized management algorithms including measurement of lactate	High	Strong
	Prompt antibiotic administration should occur in line with existing international guidelines on sepsis management when signs of sepsis are present, or when the underlying surgical pathology makes the patient at high risk of infection or sepsis such as patients with peritonitis or hollow viscus perforation. Specific antibiotic choice should be guided by local protocols in line with antimicrobial stewardship. Delay to antibiotic administration in patients with sepsis increases mortality	High	Strong
	Monitoring of blood lactate as a marker of risk and in monitoring of response to resuscitation should be considered even in the absence of sepsis	High	Strong
3. Early imaging, surgery, and source control of sepsis	Delay to surgery increases mortality in patients with sepsis and septic shock. All patients with septic shock should receive source control with surgery or interventional radiology as soon as possible and within 3 h. For patients with sepsis without septic shock source control should occur within 6 h	High	Strong
	Perform a CT scan with IV contrast as soon as possible if indicated. The CT scan should be reviewed by a radiologist immediately. Acquiring a CT scan should not cause a delay to surgery if surgery is very urgent	High	Strong
4. Risk assessment	A risk score using a validated model should be performed on all patients prior to surgery and at the end of surgery. The score can be used to guide pathways of care and facilitate discussion between team members, and with patients and family on treatment, risks and limitations	High	Strong
5. Age-related evaluation of frailty, and cognitive assessment	All patients over 65 years of age, and others at high risk, for example, patients with cancer, should be assessed for frailty using a validated frailty score	High	Strong
	Perform a validated simple assessment of cognitive function such as the Mini-Cog® in all patients over 65 years of age if time permits. For patients who are at risk for delirium and postoperative cognitive dysfunction take steps to keep the patient oriented and avoid drugs known to cause harm as defined in the Beers’ criteria	Moderate	Strong
	All patients over 65 should have regular delirium screening pre and postoperatively with a validated assessment method	High	Strong
	Patients over 65 years of age should be assessed by a physician with expertise in care of the older patient (geriatrician) preoperatively and evidence-based elder-friendly practices used. If preoperative assessment is not possible refer for postoperative follow-up	Low	Strong

ERAS item	Guideline	Level of evidence	Recommendation grade
6. Reversal of antithrombotic medications	Strongly consider reversal of home anticoagulation medications when major surgical intervention is planned. This decision should be based on both the patient’s risk of procedure-related bleeding and the risk of thromboembolism	Moderate	Strong
	Consider platelet transfusion in patients taking antiplatelet therapy when the planned procedural bleeding risk is high. In patients with a strong indication for antiplatelet therapy, specialty consultation should be obtained for perioperative co-management of these medications	Low	Weak
7. Assessment of venous thromboembolism risk	Patients should be risk assessed with a validated tool for VTE risk on admission. If pharmaceutical prophylaxis is not possible mechanical prophylaxis should be used. Reassessment should occur daily postoperatively	High	Strong
8. Pre-anesthetic medication – anxiolysis and analgesia	Sedative medication should be avoided preoperatively to avoid the risk of micro-aspiration, hypoventilation and delirium	Moderate	Strong
	Analgesia should be given to alleviate the patient’s pain and stress	High	Strong
	Multi modal opioid-sparing analgesia should be titrated to effect to maximize comfort and minimize side-effects	High	Strong
9. Preoperative glucose and electrolyte management	Hyperglycemia and hypoglycemia are risk factors for adverse postoperative outcomes. Preoperatively, glucose levels should be maintained at 144-180 mg/dL (8-10 mmol/L), a variable rate (sliding scale) insulin infusion should be used judiciously to maintain blood glucose in this range with appropriate monitoring of point of care blood glucose in line with local protocols, to avoid hypoglycemia	Moderate	Weak
	Correction of potassium and magnesium prior to surgery should be done using the intravenous route with appropriate monitoring and following local hospital policy. However, it should not delay the patient being taken to the operating room	Moderate	Weak
10. Preoperative carbohydrate loading	Authors could not recommend use of preoperative carbohydrate loading in the emergency laparotomy population		
11. Preoperative nasogastric intubation	Preoperative nasogastric tube insertion should be considered on an individual basis assessing for the risk of aspiration and gastric distension depending on the pathology and patient factors	Moderate	Strong
12. Patient and family education and shared decision making	Patients and families should have the opportunity to discuss the risk of surgery with a senior physician (this could be the surgeon, anesthesiologist or intensive care physician) prior to surgery. Counselling should be informed by a validated risk score but with the clear understanding that scores have limitations when applied to individual patients. When appropriate, treatment escalation plans and advance care plans should be discussed and documented	Low	Strong
	Clear, concise, written information or decision aids combined with verbal patient education should be provided to the patient and family before surgery if possible	Low	Strong

### Preoperative phase

The preoperative phase of an ERAS protocol for emergency laparotomy aims to rapidly correct alterations in the patients’ physiologic homeostasis. Management of physiological derangement should occur alongside investigation and diagnosis. Surgery is a key component to correction of the underlying pathology and when appropriate should occur without delay. The following evidence-based components should be incorporated into a preoperative pathway of care for each patient undergoing emergency laparotomy. Organization of care to ensure these key components are delivered reliably, by highly skilled personnel, to all patients regardless of location and type of presentation is one of the main challenges to improving care for these high-risk patients. [25–28, 33, 36].

#### 1. Early identification of physiological derangement, and intervention

ERAS protocols are designed to minimize the physiological impact and stress response of the surgical insult. For patients who require emergency laparotomy the insult and physiological derangement driven by inflammation, surgical stress and decompensation, are already occurring prior to the surgery. Resuscitation must go hand in hand with diagnostic interventions and preparation for surgery. Optimization consists of two parts: (1) patient optimization and (2) system optimization regarding availability of facilities and efficient care pathways [45]. Physiological derangements at presentation include a marked stress response, gut dysfunction, insulin resistance, fluid shifts, SIRS and sepsis with varying degrees of organ dysfunction [12, 30]. Emergency surgical patients may be hypovolemic with a potential critical impact on renal function and circulation. These derangements require early recognition and management with a sense of urgency. There is little evidence supporting delay for optimization prior to surgery in this patient category [1, 3, 17, 29, 46]. Some cohort studies have used standardized perioperative care protocols including screening and management of sepsis in line with the Surviving Sepsis Campaign [46] such as initial circulatory and respiratory stabilization, early goal-directed fluid therapy, thorough and invasive monitoring of vital parameters, and minimization of surgical delay. These studies have shown a reduction in mortality [1, 3, 6, 29]*.* Preoperative goal-directed fluid therapy was used in two of the multimodal cohort studies which showed significant reduction in mortality [3, 29]. Another small study used a goal-directed fluid optimization protocol in the preoperative holding room in patients with perforated peptic ulcer and showed reduced length of stay and mortality compared with a usual management control group [47]. Lactate guided resuscitation of patients with septic shock has been shown to reduce mortality [46] and may be beneficial in patients undergoing emergency laparotomy. The issue is not whether to delay for optimization but rather that staff competent in the management of significant physiological derangement must be involved at the earliest possible stage.

The pathophysiological abnormalities vary depending on the patient’s underlying health and co-morbidities, metabolic and immune status [48], and the underlying disorder and the duration of injury before presentation [27, 30, 36]. Patients who undergo emergency laparotomy represent only a fraction of the total volume of emergency general surgical cases but constitute the overwhelming majority of cases resulting in mortality and morbidity [32]. Use of physiological track and trigger systems [49] such as Early Warning Scores have been found to be highly predictive for severity of outcome including ICU admission and mortality in emergency surgical patients [50]. Scoring systems found to be predictive in emergency surgical patients include the Acute Physiological and Chronic Health Evaluation (APACHE II), Physiological and Operative Severity Score for the enUmeration of Mortality (POSSUM), Portsmouth-POSSUM (P-POSSUM), Modified Early Warning Score (MEWS) and National Early Warning Score (NEWS) (UK) [51]. In particular deteriorating early warning scores, in comparison to stable or improving scores, are highly predictive of mortality [52].

Recommendations:

- Resuscitation and correction of underlying physiological derangement should begin immediately and should continue during diagnostic pathways.
- Rapid assessment of the patient for physiological derangement using a validated method [49] such as an Early Warning Scoring (EWS) system should occur. Abnormal scores should trigger rapid escalation to senior personnel in line with pre-established local protocols. While awaiting surgery patients should have regular re-evaluation, with a frequency dictated by local physiological track and trigger protocols.

- Level of evidence: High.
- Recommendation grade: Strong.

#### 2. Screen and monitor for sepsis and accompanying physiological derangement

The presence of sepsis should be considered in all patients undergoing emergency surgery at presentation. One large prospective study found an incidence of over 20% of sepsis or septic shock in patients presenting to an emergency general surgical service [53]. Upregulation of the inflammatory response as occurs with SIRS and sepsis is a major contributor to death; one major study found an increased hazard ratio of death in emergency surgical patients of 1.9 for those with SIRS, and 6.7 for patients with septic shock [54]. A study of 360,000 general surgical patients from the NSQIP database found that the presence of any comorbidity increased the risk of sepsis and septic shock six-fold, and increased 30-day mortality 22-fold [55]. The three major risk factors for sepsis and septic shock were age > 60 years, comorbidity and emergency surgery. The authors commented that these patients would benefit from mandatory sepsis screening in order not to miss the window of early intervention in which the septic source must be eliminated, and physiologic derangements corrected. The presence of hypotension secondary to sepsis has a particularly poor outcome [55–58], with one large study of perforated peptic ulcer showing a 6% increased odds of 90-day mortality on adjusted risk analysis *per hour delay* to surgery in patients with preoperative hypotension [57]. Clinicians should have a high index of suspicion of sepsis when assessing emergency surgery patients.

The Sepsis 3 guidelines [59] recommend the use of quick Sepsis-related Organ Failure Assessment (qSOFA) as a screening tool to identify patients who are at risk of developing sepsis and septic shock. A positive qSOFA score should prompt further investigation for organ dysfunction, to initiate or escalate therapy as appropriate, and to consider referral to critical care or increase the frequency of monitoring. The qSOFA score may have limitations for emergency surgery patients and the use of the EWS to identify deterioration due to sepsis outperformed SIRS and qSOFA score in one large study [51]. In a review of sepsis-screening tools for surgery, it was noted that signs of sepsis in surgical patients may be diffuse and there is no perfect screening tool—what is clear is that when screening tools are used there is increased recognition of sepsis [60].

**Fig. 1:**
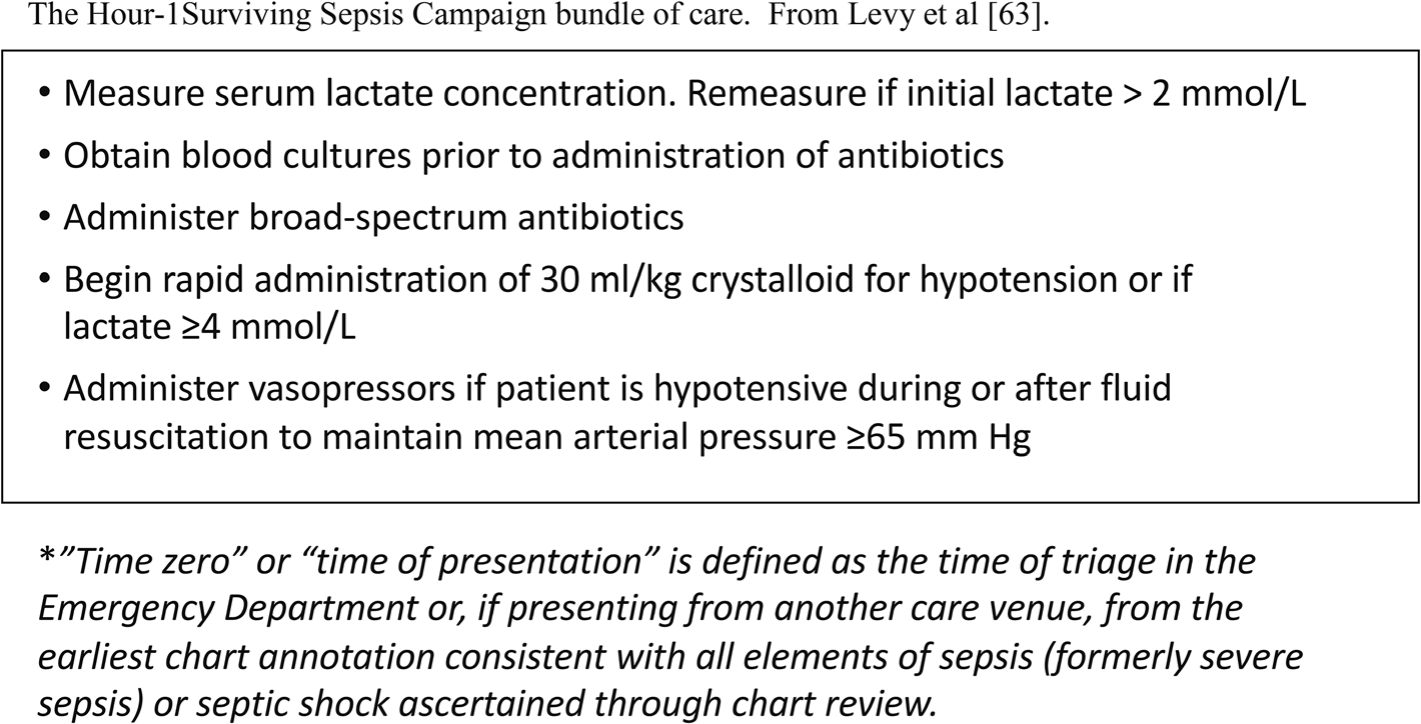
The hour-1 surviving sepsis campaign bundle of care. From Levy et al. [63]

Once sepsis is suspected clinically, validated management algorithms should be completed with a sense of urgency [46, 61, 62]. These algorithms all include the empiric administration of broad-spectrum antibiotics (after relevant cultures have been obtained when possible), and cardiovascular resuscitation using intravenous fluids titrated to clinical endpoints. Specific antibiotic choice should be guided by local protocols in line with antimicrobial stewardship. Further evaluation and escalation should also follow these algorithms (Fig. 1). The NELA 2019 report found that there was major room for improvement in speed and urgency of management with only 19% of patients with suspected sepsis receiving antibiotics within the first hour [17]. Studies have shown an association between early risk scoring, active management and a reduction in mortality [1, 17]. Blood lactate has been used as a marker of risk [63], and in monitoring of response to resuscitation in line with the Surviving Sepsis guidelines [46, 63].

Recommendations:

- All patients for emergency laparotomy should be assessed with a validated sepsis score as early in their presentation as possible. This should be repeated at appropriate intervals in line with severity of signs and sepsis risk stratification guidance [61, 63].
- If SIRS, sepsis or septic shock are diagnosed, or when the underlying surgical pathology makes the patient at high risk of infection or sepsis, such as patients with peritonitis or hollow viscus perforation, treatment should begin immediately in line with the Surviving Sepsis management algorithms including measurement of lactate [46]. Delay to antibiotic administration in patients with sepsis increases mortality [64].
- Monitoring of blood lactate as a marker of risk and in assessment of physiological response to resuscitation should be considered even in the absence of sepsis.

- Level of evidence: High-large prospective cohort studies and international guidelines.
- Recommendation grade: Strong.

#### 3. Early imaging, surgery, and source control of sepsis

Delay to surgical intervention can be due to any or all of the following: delayed diagnosis, preoperative therapeutic interventions/optimization or logistics. However, delay for patients undergoing emergency laparotomy can lead to increased mortality. In a Danish National cohort of perforated peptic ulcers, there was a 2.4% decreased probability of survival for every hour delay from hospital admission [65]. A large UK study of perforated peptic ulcer found that for patients in shock there was an increase of 6% in risk-adjusted odds of death for every hour laparotomy was delayed after admission [57]. A small Japanese study of perforation with septic shock found no patients survived to 60 days if surgery was delayed more than 6 h [66]. In another Danish cohort of all high-risk emergency laparotomy patients including those with obstruction as well as perforation, no statistically significant adjusted association between delay and surgical mortality at 90 days was found [67]. In a study from the NELA database, specifically focusing on small bowel obstruction, increased mortality was found for patients delayed more than 72 h [68]. In a multi-center study of septic patients in German ICUs when source control was needed, a delay of 6 h or more was associated with increased mortality [69]. Multimodal perioperative cohort intervention studies in emergency laparotomy have all had a surgical delay target of < 6 h from time of decision to operate to surgery, and have been associated with improved outcome [1, 3, 6, 29]. However, none of these studies have analyzed the impact of delay on survival in patients with perforation *versus* patients with obstruction. While these two clinical entities comprise the majority of indications for emergency laparotomy, the initial pathophysiology may be quite different. Patients with perforations often present with sepsis and the data are very clear that there should be minimal delay to surgical intervention, while the perioperative pathophysiology of patients with obstruction is poorly researched and the impact of delay and resuscitation less clear [3, 57, 65, 66, 68, 70]. Standards for time to surgery have been set in the UK by the Royal College of Surgeons [70, 71] and monitored by NELA and have been shown to be achievable at the 80% level [17].

Recommendations:

- Delay to surgery increases mortality in patients with sepsis and septic shock. All patients with septic shock should receive source control with surgery or interventional radiology as soon as possible and within 3 h. For patients with sepsis without septic shock, source control should occur within 6 h.

- Level of evidence: High-large prospective cohort studies, large retrospective cohort studies, national guidelines.
- Recommendation grade: Strong.

##### Radiological investigation

Examination and identification of clinical signs are the main means of diagnosis of an acute abdomen. However, patients who undergo emergency laparotomy present both in the emergency department and on hospital wards, with complications secondary to primary surgery or initial misdiagnosis of their condition. NELA has shown significant delays to surgical review (up to 8 times longer) if the initial presentation is to an internal medicine team [17]. The gold standard for diagnosis is abdominal computed tomography (CT) scan with intravenous contrast, which is recommended as “usually appropriate” for all presentations of acute abdomen unless eGFR is less than 30 (mL/min/1.73 m^2^) [72]. Intravenous contrast improves diagnostic information and the risk of allergic reaction or acute kidney injury to iodinated contrast is minimal [73, 74]. CT scans allow surgeons to visualize the problem and plan an optimal approach with the goal of reducing complications. However, CT scans and other diagnostic tests can present a time delay, and optimal diagnostic pathways to minimize delay should be used [75].

Recommendation:

- Perform a CT scan with IV contrast as soon as possible if indicated. The CT scan should be reviewed by a radiologist immediately. Acquiring a CT scan should not cause a delay to surgery if surgery is very urgent.

Level of evidence: High.

Recommendation grade: Strong.

#### 4. Risk assessment

Risk assessment has become an important tool to support clinical assessment in management of patients undergoing emergency laparotomy [70]. Risk scoring was promoted in the first Higher Risk Surgical patient guidelines in the UK [71], as many laparotomy patients were not receiving care appropriate to their risk, such as planned admission postoperatively to an ICU. Clinical teams, inexperienced in management of emergency laparotomy, underestimated the potential for poor outcome. A risk score facilitates communication amongst clinical teams about priorities and pathways including for patient transfer, prompts involvement of highly experienced staff and helps direct discussion with the patient and family. There are a number of surgical or disease-specific risk prediction tools [76–80]. Some, such as the Portsmouth-POSSUM (P-POSSUM) score [77], were developed many years ago for retrospective comparison of observed and expected outcomes when all variables are known. There is some concern regarding over-interpretation of individual patient preoperative prediction when these scores are used prospectively, and some variables must be estimated. Risk prediction scores give a population risk based on a risk model, scores can over and under-estimate risk for individual patients and clinicians must be cautious in using these scores for prognosticating patient outcomes. An example is a patient with a perforated peptic ulcer, who is acutely unwell with markedly deranged physiology and a very high-risk score, but who may benefit from rapid relatively simple surgery.

The large number of patients in the NELA database and the American College of Surgeons NSQIP database have allowed development of specific risk tools for patients undergoing emergency laparotomy which more consistently predict the actual risk for high-risk patients [78, 79]. A recent review comparing the NELA tool with ACS-NSQIP, P-POSSUM and APACHE II found the NELA score showed the highest discrimination with an area under the curve (AUC) of 0.83, ACS-NSQIP had an AUC of 0.80 [81]. When a risk score was calculated retrospectively on patients in the NELA dataset who had not been risk scored preoperatively or at the end of surgery, those patients were less likely to have potentially protective perioperative interventions such as planned postoperative critical care admission, and had poorer outcomes than a risk matched cohort who had prospective risk scoring performed [17]. Having a risk score available preoperatively may facilitate communication and planning for patient care. An ethnographic study of surgical teams, particularly those who did not routinely manage emergency surgical patients, found that use of a score enhanced inter-professional communication and decision making [82].

Most risk prediction tools do not directly adjust for certain relatively uncommon co-morbidities and some specific acute abdominal pathologies (other than cancer) which probably impact significantly on the survivability of an emergency laparotomy. Examples include a patient with a severe neurological condition or a patient with bowel infarction. When applying a risk prediction tool to an individual patient it is prudent to consider whether there are additional risk-altering variables present that have not been taken into account in any calculation. Additionally, these models are derived from patients who underwent surgery and do not include those evaluated for surgery but declined secondary to prohibitive risk.

Recommendation:

- A risk score using a validated model should be performed and documented on all patients prior to surgery, and at the end of surgery. The score can be used to guide pathways of care and facilitate discussion between team members and with patients and family on treatment, risks and limitations.

- Level of evidence: High.
- Recommendation grade: Strong.

#### 5. Age-related evaluation of frailty, and cognitive assessment

Age and frailty are not the same. Frailty is defined as an age-related state of decreased physiologic reserve [83]. All the large studies show that age alone is significantly associated with poor outcomes for emergency laparotomy [14]. In NELA mortality for patients over 80 years old in particular, remains very high at 17% at 30 days and 22% at 90 days [17]. Clearly the risk for these patients is so high that if emergency surgery is to be performed meticulous delivery of all evidence-based pathway components is essential. An ERAS approach has been shown to reduce mortality in patients over the age of 70 [1, 6]. A systematic review found an ERAS approach to be beneficial for older patients undergoing emergency surgery [7]. Many of these patients will also be frail, resulting in a lack of resilience in the face of a physiological insult [84–87], and a validated frailty assessment [70, 83, 88, 89] should be performed if possible acknowledging the limitations in the acute environment. Frail patients and those with cognitive impairment have a higher risk of mortality and morbidity which may not be captured by the commonly used surgical risk scores [70, 81, 90]. In a study of outcomes at 12 months in older patients after emergency laparotomy, the strongest predictors of mortality were frailty and increased American Society of Anesthesiologists (ASA) status [91]. Involvement of a physician specialized in the care of older adults to co-manage these patients, and/or the use of targeted interventions should occur as soon as possible and is associated with better outcomes. The strongest evidence for comprehensive geriatric assessment exists for patients with hip fractures [92–95], but a recent paper shows postoperative geriatrician review was associated with reduced mortality in patients over the age of 65 years undergoing emergency laparotomy [26, 96]. Another recent study using a proactive approach for patients over 65 years presenting for emergency general surgery with integration of a geriatric assessment team, optimization of evidence-based elder-friendly practices, promotion of patient-oriented rehabilitation, and early discharge planning found a significant reduction in mortality, length of stay and discharge to a higher level of care [95]. Proactive management of these frail patients may also decrease the costs of care [97, 98]. At present the evidence indicates that most older emergency laparotomy patients are not reliably assessed for frailty nor co-managed with a care of the elderly team [17].

##### Delirium and perioperative neurocognitive disorders

Patients over 65 years of age who undergo emergency surgery are at particular risk for delirium and perioperative neurocognitive disorders [99–101]. All patients over 65 years of age and any with risk factors for preexisting cognitive impairment should have a baseline assessment of cognition with a simple screening tool [102–105] and a preoperative assessment of delirium. Patients should be monitored regularly for delirium with awareness that hypoactive delirium occurs more commonly than an agitated delirious state and has a poorer outcome [106]. The American College of Surgeons and the American Geriatric Society (AGS) have joint guidelines on how to prevent, diagnose and care for delirium in the surgical patient [104]. If delirium should occur, it is associated with increased mortality, complications, readmission, and long-term cognitive decline [107, 108]. Delirium is preventable in about 40% of cases with simple steps [104, 109, 110] and avoidance of drugs that fall under AGS Beers criteria drugs, such as benzodiazepines and anticholinergics [109, 111] Table 2. Incorporation of a “hospital elder life program” with simple measures such as mouth care and regular orienting communication for patients undergoing major elective intra-abdominal surgery demonstrated a significant reduction in the incidence of delirium [110].

**Table 2:** Medications commonly given in perioperative care that should be avoided or used with caution in patients over 65 year of age adapted from the 2019 American geriatric society Beers criteria [111] and Berger et al. [103]

Medication or class of medication	Examples	Rationale for avoiding
First generation antihistamines	Diphenhydramine, Chlorpheniramine	Central anticholinergic effects
Phenothiazine-type antiemetics	Prochlorperazine, Promethazine	Central anticholinergic effects
Antispasmodics/anticholinergics	Atropine, Scopolamine	Central anticholinergic effects
Antipsychotics(first and second generation)	Haloperidol	Risk of cognitive impairment, delirium, neuroleptic malignant syndrome, tardive dyskinesia
Benzodiazepines	Midazolam, Diazepam, Temazepam	Risk of cognitive impairment, delirium
Benzodiazepine receptor agonist hypnotics “ Z drugs”	Zolpidem, Eszopiclone	Delirium, falls
Corticosteroids	Hydrocortisone, Methylprednisolone	Risk of cognitive impairment, delirium, psychosis
H_2_-receptor antagonists	Ranitidine	Risk of cognitive impairment, delirium
Metoclopramide		Extrapyramidal effects
Pethidine/Meperidine		Neurotoxic effect

H_2_, histamine 2 receptor.

Recommendations:

- All patients over 65 years of age, and others at high risk, for example patients with cancer, should be assessed for frailty using a validated frailty score [83].
  Evidence: High.
  Recommendation grade: Strong.
- Perform a validated simple assessment of cognitive function such as the Mini-Cog® [112] in all patients over 65 years of age if time permits. For patients who are at risk for delirium and postoperative cognitive dysfunction take steps to keep the patient oriented and avoid drugs known to cause harm as defined in the Beers’ criteria [111].
  Level of evidence: Moderate.
  Recommendation grade: Strong.
- All patients over 65 should have regular delirium screening pre and postoperatively with a validated assessment method [113].
  Level of evidence: High.
  Recommendation grade: Strong.
- Patients over 65 years of age should be assessed by a physician with expertise in care of the older patient (geriatrician) pre-operatively and evidence-based elder-friendly practices used. If preoperative assessment is not possible refer for postoperative follow-up.

- Level of evidence: Low.
- Recommendation grade: Strong.

#### 6. Reversal of antithrombotic medications

##### Anticoagulants and platelet function inhibitors

Long-term antiplatelet and anticoagulation use is increasingly common in many populations, and their management in emergency surgery is complex. Patients undergoing emergency laparotomy are at high risk of perioperative hemorrhage and thrombosis, given both the nature of their procedure and the underlying coagulopathy of sepsis and systemic inflammation [114]. Hemorrhage following surgery is highly-associated with end-organ dysfunction and mortality in emergency general surgical patients [115–119]. Reversal of these agents or their effects prior to emergent surgery, when possible, may reduce the risk of perioperative hemorrhage. Vitamin K antagonists such as warfarin are common, although newer direct-acting oral anticoagulants (DOACs) are increasingly used [120]. Guidance on reversal of specific antithrombotic medications and platelet function inhibitors has been published by various societies and is beyond the scope of this article [121–124]. To guide management decisions, coagulation tests including international normalized ratio (INR), prothrombin time (PT), and partial thromboplastin time (PTT) among others should be obtained preoperatively where appropriate, although conventional clotting studies do not help with low molecular weight heparin or DOACs. A variety of platelet function tests are available and should be considered for patients taking antiplatelet therapy [124, 125]. The decision to reverse antithrombotic medication should be based on the patient’s immediate need for surgery, the risk of associated bleeding, and the risk of thromboembolism [121, 122].

##### Anticoagulants (Warfarin, DOACs, Heparin/Enoxaparin)

Prothrombin complex concentrate (PCC) and fresh frozen plasma may be administered and titrated to the required effect in these cases [121, 122]. Specific reversal agents exist for DOAC medications and can be used if available. Dabigatran has a reversal agent idarucizumab [126] and recombinant factor Xa functions to reverse apixaban and rivaroxaban [127].

Recommendation:

- Strongly consider reversal of home anticoagulation medications when major surgical intervention is planned. This decision should be based on both the patient’s risk of procedure-related bleeding and the risk of thromboembolism.

- Level of evidence: Moderate.
- Recommendation grade: Strong.

##### Platelet inhibitors: (including Aspirin, Clopidogrel, Dipyridamole, Ticagrelor)

Patients taking antiplatelet medications may be considered for platelet transfusion if warranted given the risk of bleeding from the planned operation. There is some evidence that transfused platelets may partially reverse the effects of these medications [122, 128]. If a patient is taking only aspirin at the time of surgery, many surgeons elect to proceed without reversal [121, 129]. Patients taking dual antiplatelet therapy are likely at higher risk of bleeding complications and transfusion [125, 130]. P2Y12 and aspirin response assays are available in some hospitals to assess the impairment of platelet function by these drugs. If the risk of surgical delay is high, a reasonable approach may be to proceed to surgery and transfuse platelets if excess bleeding is encountered [125]. For patients with recent coronary artery stenting, given the risk of adverse cardiac events consultation and co-management of antiplatelet therapy with a cardiologist is recommended.

Recommendation:

- Consider platelet transfusion in patients taking antiplatelet therapy when the planned procedural bleeding risk is high. In patients with a strong indication for antiplatelets, specialty consultation should be obtained for perioperative co-management of these medications.

- Level of evidence: Low.
- Recommendation grade: Weak.

#### 7. Assessment of venous thromboembolism risk

Compared with elective surgical patients undergoing a comparable intra-abdominal procedure, emergency patients are at increased risk of venous thromboembolism (VTE) [131, 132]. Patients should be assessed for risk with a validated tool at admission, and VTE prophylaxis (mechanical and/or pharmacologic) should be initiated as soon as possible even if surgery is planned [131, 133, 134]. If pharmaceutical prophylaxis is not an option, mechanical prophylaxis should be used. The patient should be reassessed at regular intervals pre and postoperatively [131].

Recommendation:

- Patients should be risk assessed with a validated tool for VTE risk on admission. If pharmaceutical prophylaxis is not possible mechanical prophylaxis should be used. Reassessment should occur daily postoperatively [131].

- Level of evidence: Strong (extrapolated from studies in elective major abdominal surgery).
- Recommendation grade: Strong.

#### 8. Pre-anesthetic medication—anxiolysis and analgesia

Patients with an acute abdomen often require strong analgesia. Pain should be assessed, and an appropriate intervention should be made using multimodal analgesic titration and by minimizing the amount of opioid used to achieve effective analgesia. Opioids increase the risk of a patient being over-sedated, hypo-ventilating and even aspirating, so appropriate monitoring should be performed. The addition of benzodiazepines or other sedative agents compound these risks and should be avoided, and can increase the risk of postoperative delirium in older patients [106]. The use of preoperative nerve blocks such as transversus abdominis plane (TAP) blocks prior to surgery do not address the peritoneal and visceral pain of an acute abdomen. Therefore, opioids are often necessary in addition to other multimodal agents. Non-steroidal anti-inflammatory drugs (NSAIDs) are best avoided, due to the high risk of acute kidney injury (AKI) in this population, until postoperatively when renal function has normalized [44, 135, 136].

Recommendations:

- Sedative medication should be avoided preoperatively to avoid the risk of micro-aspiration, hypoventilation and delirium.

- Evidence: Moderate.
- Recommendation grade: Strong.

- Analgesia should be given to alleviate the patient’s pain and stress.

- Evidence: High.
- Recommendation grade: Strong.

- Multi modal opioid-sparing analgesia should be titrated to effect to maximize comfort and minimize side-effects.

- Evidence: High.
- Recommendation grade: Strong.

#### 9. Preoperative glucose and electrolyte management

Perioperative glucose control is important to maintain body homeostasis and reduce downstream complications [137]. Hyperglycemia during and after surgery is common, occurring in 20–40% of elective surgical patients, particularly in diabetic patients or those with impaired glucose tolerance. Elevated blood glucose impairs neutrophil function and can cause overproduction of inflammatory mediators, reactive oxygen species and free fatty acids causing direct cellular damage, vascular endothelial changes and immune dysfunction. Substantial evidence indicates that correction of hyperglycemia with insulin administration reduces hospital complications and decreases mortality in general surgery patients [137–139]. The stress response drives insulin resistance at a time when patients are likely to have poor oral calorie intake and omit their insulin or diabetic tablets for fear of hypoglycemia. ERAS pathways for elective surgery try to mitigate this insulin resistance by using components such as oral carbohydrate loading and regional anesthesia [44]. However, this is usually not feasible in emergency laparotomy patients.

A proactive approach to avoid both hyper and hypoglycemia should be taken in emergency laparotomy patients. Pre-operative blood glucose levels should be controlled in a similar range to critical care patients—between 144–180 mg/dL (8–10 mmol/L). Tight control of blood sugar has been relaxed since the first tight glycemic control ICU studies, the incidence of complications appears not to be significantly altered when allowing blood glucose to be 180 mg/dL (10 mmol/L) [140] but with a reduction in hypoglycemic neurological complications [141]. Most patients will be taking minimal calories by mouth and be receiving intravenous resuscitation and ongoing maintenance fluid with balanced crystalloid infusions which contain no glucose. Hypoglycemia should be treated with an intravenous 50% dextrose bolus and appropriate follow-up dextrose administration, again according to local hospital policy. It is unclear for hyperglycemia, whether a basal-bolus of insulin [138] is beneficial compared with a standard sliding scale in the emergency surgical population. Judicious use of a variable rate insulin infusion (sliding scale) regimen should be utilized according to local hospital policy and attention given to plasma potassium levels that can be lowered by insulin administration. The ongoing management of glucose control is outlined in Part 2 of this Guideline. An HbA1c taken on admission is useful in guiding whether a patient has long-term glycemic control issues and may aid decision making on clinical intervention.

Electrolyte disturbance is common in this group of patients due to high fluid shifts and external losses of body fluids. Hypokalemia, hypomagnesemia and hypophosphatemia are risk factors for cardiac dysrhythmias, particularly atrial fibrillation which is particularly common in this patient group due to age and preexisting atrial fibrillation, fluid shifts, electrolyte imbalance and septic shock needing vasopressor infusions. Attempts should be made to correct low potassium, phosphate and magnesium using intravenous repletion with appropriate monitoring and according to local policy to reduce the risk of atrial fibrillation [142].

Recommendations:

- Hyperglycemia and hypoglycemia are risk factors for adverse postoperative outcomes. Pre-operatively, glucose levels should be maintained at 144–180 mg/dL (8–10 mmol/L), a variable rate (sliding scale) insulin infusion should be used judiciously to maintain blood glucose in this range with appropriate monitoring of point of care blood glucose in line with local protocols to avoid hypoglycemia.
- Correction of potassium, magnesium and phosphate prior to surgery should be done using the intravenous route with appropriate monitoring and following local hospital policy. However, it should not delay the patient from being taken to the operating room.

- Level of evidence: moderate (inconsistency, extrapolated, uncertain target glucose values, potassium and magnesium extrapolated from cardiac and critical care data).
- Recommendation grade: weak (benefit must be outweighed against the risk of hypoglycemia, diabetic patients likely to benefit the most, the risk of atrial fibrillation may be reduced by fluid and electrolyte correction, but the cause is multifactorial).

#### 10. Preoperative carbohydrate loading

An oral carbohydrate drink given preoperatively is a recommendation in most other ERAS Society Guidelines to reduce dehydration and improve insulin sensitivity by giving a carbohydrate load 2–4 h prior to surgery. Emergency laparotomy patients are already under physiological stress and giving carbohydrates in this setting may elevate glucose levels further with no effect on insulin sensitivity. We could not identify any studies on the use or benefit of carbohydrate loading in emergency general surgery. The increased risk of gastric stasis, intra-abdominal pathology, preoperative use of opioids and generalized practice of using preoperative nasogastric tubes and avoiding oral intake prior to surgery meant we extrapolated evidence of potential harm and this group could not recommend the use of carbohydrate loading [44].

Recommendation:

- Level of evidence: Low and potential harm.
- Recommendation grade: Strong, do not use in the emergency laparotomy population.

#### 11. Pre-operative nasogastric intubation

Nasogastric tubes (NGTs) have been traditionally used in emergency surgery to reduce gastric distension and drain gastric contents. The use of nasogastric tubes in elective colorectal surgical patients is declining as the evidence base has shown an increase in complications such as respiratory infections and pharyngolaryngitis as well as patient discomfort and delay to feeding, [143, 144] with no change in morbidity or mortality. [145]. The use in the emergency setting is very different with a risk–benefit ratio depending on the clinical circumstances and cause of abdominal pathology and patient factors. Patients may have pathology causing gastric distension and high gastric fluid volumes, and decompression may be beneficial and reduce the risk of aspiration at induction of anesthesia. This risk benefit is different in the preoperative and postoperative setting. We therefore discuss the postoperative use and continuation in part 2 of this guideline.

Recommendations:

- Pre-operative nasogastric tube insertion should be considered on an individual basis assessing for the risk of aspiration and gastric distension depending on the pathology and patient factors.

- Level of evidence: Moderate (extrapolation from elective surgery).
- Recommendation grade: Strong (aspiration can be life-threatening and its reduction by nasogastric insertion outweighs the risk of short-term use).

#### 12. Patient and family education and shared decision making

Patient education is a central pillar of elective enhanced recovery pathways, benefits include reduced pain and anxiety [146–148]. There is less time for education or explanation of complex surgery in the emergency setting, although handouts can be given to patients and families to read before or after surgery. In very high-risk patients, surgery should not be undertaken without discussion about ceilings of care, even though this is challenging in the acute situation. Objective mortality scores can support conversations and should be used in combination with other assessments such as frailty scores [70, 149]. Shared decision making (SDM) and personalization of care is especially challenging in a patient in pain and with acute physiological disturbance from abdominal pathology [150]. Additionally, there is less time to develop the clinician-patient rapport/relationship which SDM relies upon [151]. Scenario planning and the use of decision aids may support SDM, helping to move detailed, complex conversations toward more rapidly understood and patient-centered information [152]. Using the BRAN methodology (‘benefits, risks, alternatives, do nothing) or best/worst case scenario may support a clear structured standardized approach [149, 150, 153]. Discussions should not just be about life or death, but loss of independence, quality of life and other important factors to patients, such as long-term stoma formation [154]. There is guidance available to surgical teams to help manage these situations [155] although the complexity and acuity of the situation means that eliciting patient preference and achieving goal- concordant care is challenging [156]. The goal should be to achieve active joint and realistic decision making before surgery. For all patients and families, satisfaction with emergency abdominal surgery is associated with receiving sufficient information about the risks and benefits of surgery [20, 149, 157], and it is feasible to collect patient reported outcome measures from patients who have undergone emergency laparotomy [158].

Recommendation:

- Patients and families should have the opportunity to discuss the risk of surgery with a senior physician (this could be the surgeon, anesthesiologist or intensive care physician) prior to surgery. Counseling should be informed by a validated risk score but with the clear understanding that scores have limitations when applied to individual patients. When appropriate, treatment escalation plans and advance care plans should be discussed and documented.
- Clear, concise, written information or decision aids combined with verbal patient education should be provided to the patient and family before surgery if possible.

- Level of evidence: Low.
- Recommendation grade: Strong (improves informed consent process).

## Conclusions

These guidelines present the current evidence base and recommendations for the preoperative phase of an ERAS approach to patients undergoing emergency laparotomy. Such an approach has been shown to improve outcomes for these very high-risk patients. An increased awareness of the specific risks of older patients with attention to perioperative neurocognitive disorders, frailty and geriatric care is a new addition to ERAS guidelines. The evidence base is low in certain areas and much has been extrapolated from elective ERAS guidelines and other evidence based on planned surgery. While it would be ideal to test all elements in the emergency situation, the lack of randomized controlled trials in this patient group demonstrates the challenges of research in this area. Other concepts relevant to the care of the emergency laparotomy patient such as organization of surgical services, end-of-life care and other ERAS elements will be covered in Part 2 of these guidelines. It is hoped that these pre-operative guidelines, when paired with the intra, and postoperative guidelines will provide a framework for improved management of patients undergoing emergency laparotomy.
